# 2553 Authors’ perceptions of the interdisciplinarity of their research

**DOI:** 10.1017/cts.2018.49

**Published:** 2018-11-21

**Authors:** Christine M. Weston, Mia S. Terkowitz, Daniel E. Ford

**Affiliations:** Johns Hopkins University School of Medicine

## Abstract

OBJECTIVES/SPECIFIC AIMS: The objectives of this study were to compare different methods for determining the disciplines involved in a research article. We sought to address the following questions: To what extent does the number of disciplines reported by an article’s corresponding author agree with their description of the article as unidisciplinary or interdisciplinary? (Q1) and To what extent does the corresponding author’s description of the research as unidisciplinary or interdisciplinary agree with its classification as unidisciplinary or interdisciplinary based on the affiliation of its co-authors? (Q2). METHODS/STUDY POPULATION: Using Scopus, we randomly selected 100 articles from 2010 and 2015 from science teams that had at least 1 author affiliated with Johns Hopkins. Author affiliations were grouped into common academic disciplines: Basic Science, Medicine (and all clinical specialties), Public Health, Engineering, Social Science, Computer Science, Pharmacy, Nursing, and Other. Articles with more than 1 discipline were considered, interdisciplinary. We then sent an online Qualtrics survey to the corresponding author of each article and asked them to indicate (1) all of the disciplines that contributed to the research article at hand, and (2) to indicate whether they considered the research to be “unidisciplinary” or “interdisciplinary” based on definitions that we provided. RESULTS/ANTICIPATED RESULTS: For Q1, we asked corresponding authors to indicate the number of disciplines involved in their research and then to choose the definition that best described their research. Among 76 respondents, 42 indicated that their research consisted of 1 discipline, and 34 indicated that their research consisted of more than 1 discipline. Of the 42 respondents who indicated that their research consisted of one discipline, 21 (50%) respondents described their research as “unidisciplinary” and 21 (50%) described their research as “interdisciplinary.” However, of the 34 respondents who indicated that their research consisted of more than 1 discipline, all but 1 (97%) described their research as “interdisciplinary.” For Q2, we assigned a discipline to each co-author based on his/her affiliation and counted the number of disciplines involved. Among 76 respondents, of the 22 who described their research as “unidisciplinary,” 16 (73%) were categorized as “unidisciplinary” and 6 (27%) were categorized as “interdisciplinary,” using this method. Of the 54 respondents who described their research as “interdisciplinary,” 30 (56%) were categorized as “interdisciplinary” and 24 (44%) as “unidisciplinary.” DISCUSSION/SIGNIFICANCE OF IMPACT: Our results highlight that different methods for determining whether a given research article is interdisciplinary are likely to yield different results. Even when researchers indicate that their research is based within one major discipline, they may still consider it interdisciplinary. Likewise, classifying an article as either unidisciplinary or interdisciplinary based on the affiliations of its co-authors, may not be consistent with the way it is viewed by its authors. It is important to acknowledge that assessing the interdisciplinarity of research is complex and that objective and subjective views may differ.

